# Allogeneic versus autogenous shell technique augmentation procedures: a prospective-observational clinical trial comparing surgical time and complication rates

**DOI:** 10.1186/s40729-023-00505-y

**Published:** 2023-12-20

**Authors:** Jochen Tunkel, Frederik Hoffmann, Yannik Schmelcher, Anita Kloss-Brandstätter, Peer W. Kämmerer

**Affiliations:** 1 Private Practice for Oral Surgery and Periodontology, Königstraße 19, 32545 Bad Oeynhausen, Germany; 2 Department of Engineering & IT, Carinthia University of Applied Sciences, Europastraße 4, 9524 Villach, Austria; 3 Department of Oral and Maxillofacial Surgery, University Medical Center Mainz, Augustusplatz 2, 55131 Mainz, Germany

**Keywords:** Autogenous bone, Allogeneic bone, Dental augmentation, Clinical study, Surgery time, Complications

## Abstract

**Objectives:**

Autogenous and allogeneic blocks for shell augmentation of the jaw have shown comparable results. This observational clinical study aimed to compare both materials for shell augmentation concerning surgery time and intra- and postoperative complications.

**Material and methods:**

Bone augmentation with the shell technique using autogenous or allogenous bone was performed in 117 patients with segmental jaw atrophy. The primary study parameter was the surgical time, comparing both materials. Subsequently, intra- and postoperative complications were recorded.

**Results:**

Allogeneic (*n* = 60), autogenous (*n* = 52), or both materials (*n* = 5) were used. The use of allogeneic material led to a significantly shorter operation time (*p* < 0.001). A more experienced surgeon needed significantly less time than a less experienced surgeon (*p* < 0.001). An increasing number of bone shells (*p* < 0.001), an additional sinus floor elevation, and intraoperative complications also significantly increased the operation time (*p* = 0.001). Combining allogeneic and autogenous shells (*p* = 0.02) and simultaneous sinus floor elevation (*p* = 0.043) significantly impacted intraoperative complications. No correlations were found between the included variables for postoperative complications (all *p* > 0.05). In total, 229 implants were inserted after a healing time of 4–6 months, with a survival of 99.6% after a mean follow-up duration of 9 months.

**Conclusions:**

Compared to the autogenous technique, allogeneic shell augmentation has a shorter surgical time and a similar rate of intra- and postoperative complications as autogenous bone. Together with its promising clinical results, this technique can be recommended.

**Graphical Abstract:**

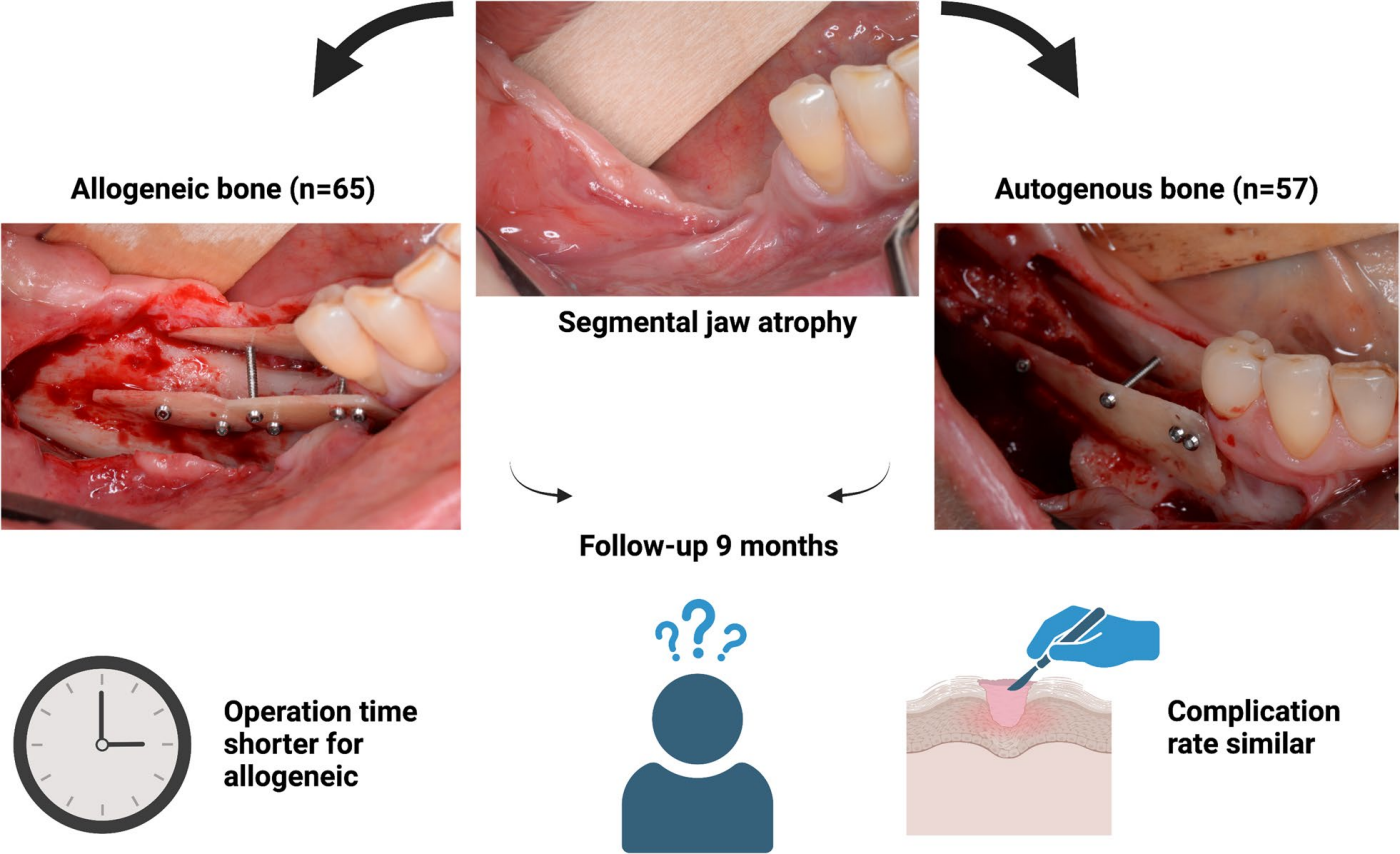

## Introduction

Based on the osseous jaw defect's localization, extent, and configuration, several augmentation techniques and surgical approaches have been established [1–3]. When using autogenous bone blocks, most patients reported their preference for the retromolar region as the donor site [4], which has been shown to have a low donor site morbidity [5]. However, some patients experienced postoperative pain during chewing, swelling episodes, and bleeding [6–8]; a particular risk of infection, mucosal dehiscences, and primarily temporary neurosensory disturbances is described [9, 10]. Allogeneic bone is a promising alternative to autogenous material for dental augmentation with clinically similar results but is readily available without any donor site morbidity [11–14]. Osteoinduction is discussed next to the osteoconductive effect of allogeneic bone, which seems to depend on the allograft processing [15]. Whereas its safety has been discussed controversially [16, 17], there are no adverse reports for mineralized processed bone allografts (DBM) that are considered safe products [12, 18, 19].

Khoury et al. made the shell technique popular [20], which can be performed for horizontal and/or vertical bone gain using autogenous or allogeneic bone [12, 21, 22] with promising results. In brief, these shells form a secluded space between the material and the residual bone that can be filled with various bone substitute materials for osseous regeneration [12, 14, 23, 24]. Even if the evidence on allogeneic blocks/shells for dental augmentation purposes is increasing over time [25, 26], Smeets et al. concluded in their systematic review on horizontal augmentation techniques in the mandible that the use of allogeneic bone blocks must be re-evaluated in an evidence-based way [1].

In the literature, a further advantage of allogeneic bone shells compared to autogenous bone obtained from the mandibular ramus is the shorter time of surgery and a subsequent decrease in infection risks as well as general donor site morbidity [10, 27]. To our knowledge, clinical trials have yet to prove this. Therefore, this non-interventional, prospective clinical trial aimed to compare the time of surgery when using allogeneic and/or autogenous shells for dental augmentation. The null hypothesis was that the two materials had no difference in time for surgery. The secondary research parameter was the occurrence of intra- and postoperative complications when using both materials.

## Material and methods

In a non-interventional clinical trial, patients who needed bone augmentation procedures (Class II–IV defect types [28]) before dental implant placement were included. Patients were excluded if they were underage, had a history of radio- and/or chemotherapy in the head and neck region, other systemic diseases contraindicating oral surgery, uncontrolled periodontal disease, therapy with bisphosphonates, diabetes mellitus, bruxism, pregnancy, and psychiatric problems. The study was approved by the Ethics Committee of the State Medical Association of Rhineland-Palatinate, Germany (Number 2018-13776 and 2022-16445) and was registered into the database of the University Medical Center Mainz (01_08-2020). It was conducted by protocol and in compliance with the moral, ethical, and scientific principles governing clinical research in the Declaration of Helsinki of 1975, as revised in 1983. Data were collected prospectively in clinical routine and evaluated retrospectively using the patients’ charts. All patients provided informed consent before therapy and before inclusion in the study.

### Patients and procedures

In total, 117 patients (mean age 56 years (minimum: 19, maximum: 84; standard deviation: 12.7) were included. Eighty-three patients (70.9%) were female, and 34 (29.1%) were male. The indications for bone augmentation are given in Table 1. The surgical procedures were carried out as described before [12]. In brief, a full-thickness flap was raised after crestal incision with or without relieving incisions, dependent on the defect's size and geometry. Augmentation procedures were conducted using the shell technique, either with allogeneic (maxgraft® cortico, 25 × 10 × 1 mm, botiss biomaterials GmbH, Zossen, Germany) or autogenous material, dependent on the patient’s informed consent and choice. If autogenous material was chosen, it was taken from the external oblique line of the mandible after crestal incision together with two vertical releasing incisions. The mucoperiosteal flap was raised, and the bone was harvested using a microsaw (Frios MicroSaw, Dentsply Sirona, Charlotte, North Carolina, United States). After completion of the osteotomy, the donor site was closed using interrupted sutures (PGA Resorba 5-0; Resorba Medical GmbH, Nürnberg, Germany). Each shell was adjusted and trimmed to the correct size; the allogeneic shells were rehydrated in 0.9% saline at room temperature for at least 10 min to increase breaking strength and flexibility [24]. Edges were smoothed using a diamond bur, and the screw holes in the shells were drilled outside the oral cavity. After placement of the shells, they were fixed with at least two adjusting screws (1 mm Microscrews; Stoma, Tuttlingen, Germany). The shell and residual bone gap was either filled with local autogenous bone or a mixture of autogenous bone and allogeneic cancellous granules (maxgraft® granules, botiss biomaterials GmbH, Zossen, Germany). The autogenous bone was collected via bone-scaping instruments. Next, a periosteal releasing incision was carried out, and the wound was closed without tension via resorbable mono-filament sutures (PGA Resorba 5-0). After four to six months of healing, screw removal and implant placement were done. Immediately after implant placement, the augmented area was relined using a thin layer of bovine bone substitute material (cerabone®, botiss biomaterials GmbH, Zossen, Germany) as described before [29]. This was covered with a collagen membrane of porcine origin (Jason® membrane, botiss biomaterials GmbH, Zossen, Germany). All surgeries were conducted either by surgeons with an experience of < 2 years or more than 5 years.

**Table 1:** Indications for osseous augmentation

Indications	Number	Percentage
Single-tooth gap	51	43.6
Free-end situation maxilla (total)	25	21.4
Free-end situation maxilla (left)	7	6
Free-end situation maxilla (right)	12	10.3
Free-end situation maxilla (both)	6	5.1
Free-end situation mandible (total)	39	33.3
Free-end situation mandible (left)	15	12.8
Free-end situation mandible (right)	18	15.4
Free-end situation mandible (both)	6	5.1
Edentulous maxilla	2	1.7

Total number of cases was *n* = 117

### Study parameters

The primary study parameter was the time of surgery (from incision to final suture), comparing allogeneic and autogenous materials. Next, intra- and postoperative complications were recorded; postoperative complications were defined to occur during the healing phase of the augmented material for four to six months until the placement of dental implants. Data on dental implant survival were collected as well.

### Statistics

Statistical analyses were performed with IBM SPSS (version 27; International Business Machines Corp., Armonk, NY, USA), for descriptive statistics of quantitative variables, mean values, and standard deviations were calculated. The data set was complete, and there were all the data. The primary outcome variable was the operation time. The following predictors and potential confounders were extracted from the patient records: gender, the experience of the surgeon, donor site, shell material, particles, simultaneous implantation, simultaneous sinus lift, and number of shells.

Pearson's Chi-squared test was applied to sets of unpaired categorical data to evaluate the likelihood that any observed difference between the sets was due to chance. Fisher's exact test was used where sample sizes were small. An independent sample t-test was used when two sets of independent and identically distributed samples were obtained, and their population means were compared. To investigate the influence of an independent variable with more than three groups on the expression of the outcome variable, a simple analysis of variance (ANOVA) was used. Only two-sided significance tests were used. A probability of error of *p* ≤ 0.05 was chosen as the threshold value. An alpha adjustment for multiple testing was not performed. The results are, therefore, explorative and descriptive. Multiple linear regression was used to explain an observed outcome variable (operation time) by several independent variables. The categorical variables were added to the model as factors. All significant variables from the univariate analyses were included in the first model. Then, all non-significant variables were removed from the multiple linear regression model stepwise.

## Results

### Augmentation procedures

**Fig. 1:**
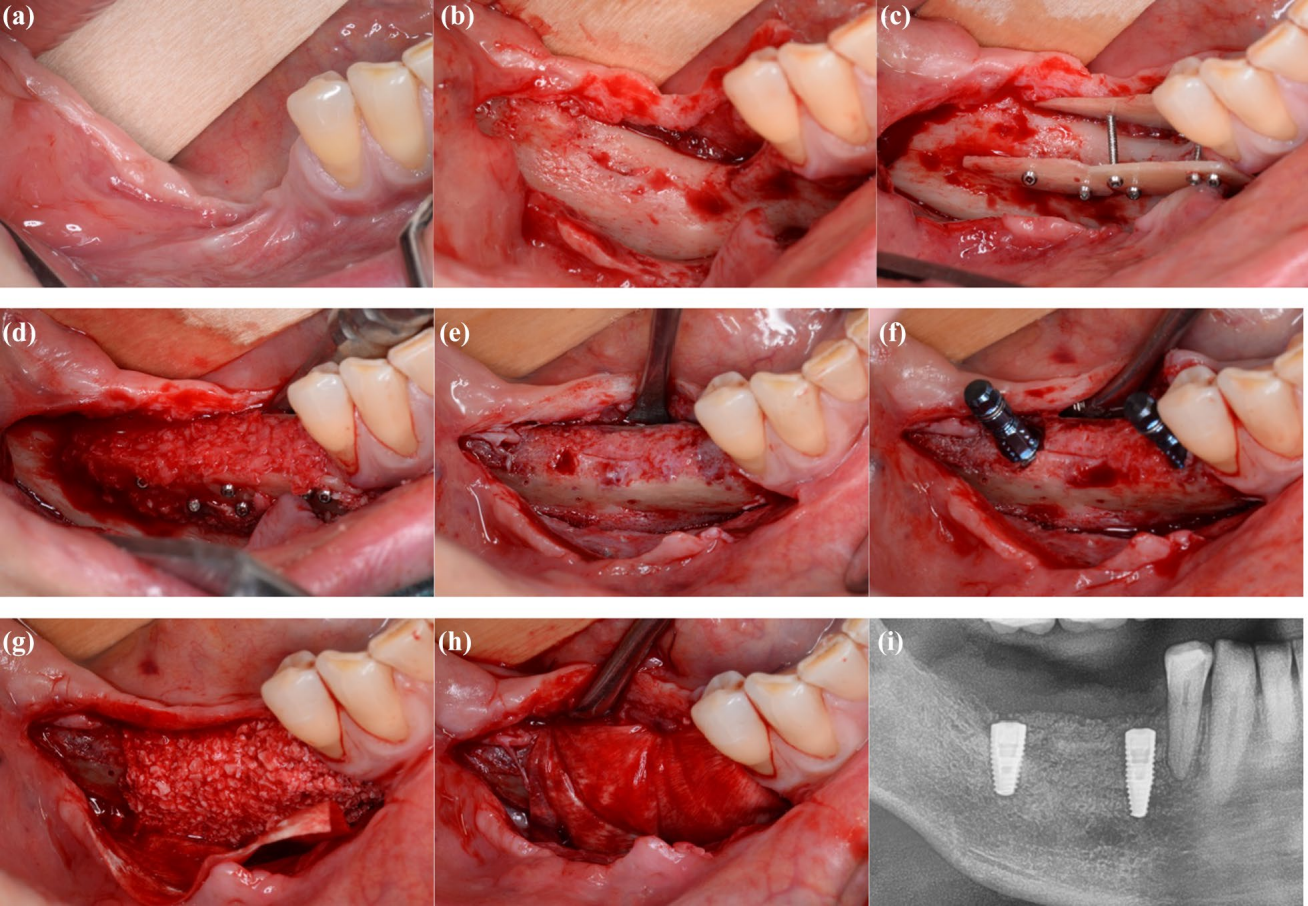
allogeneic shell technique for augmentation of the posterior mandible. **a** Preoperative site, **b** intraoperative site before augmentation, **c** placement of two allogeneic shells, **d** filling of the gap with a mixture of allogeneic and autogenous bone, **e** site after a healing time of four months, **f** placement of two dental implants, **g** relining with bovine bone, **h** coverage with a collagen membrane, **i** postoperative radiograph

**Fig. 2:**
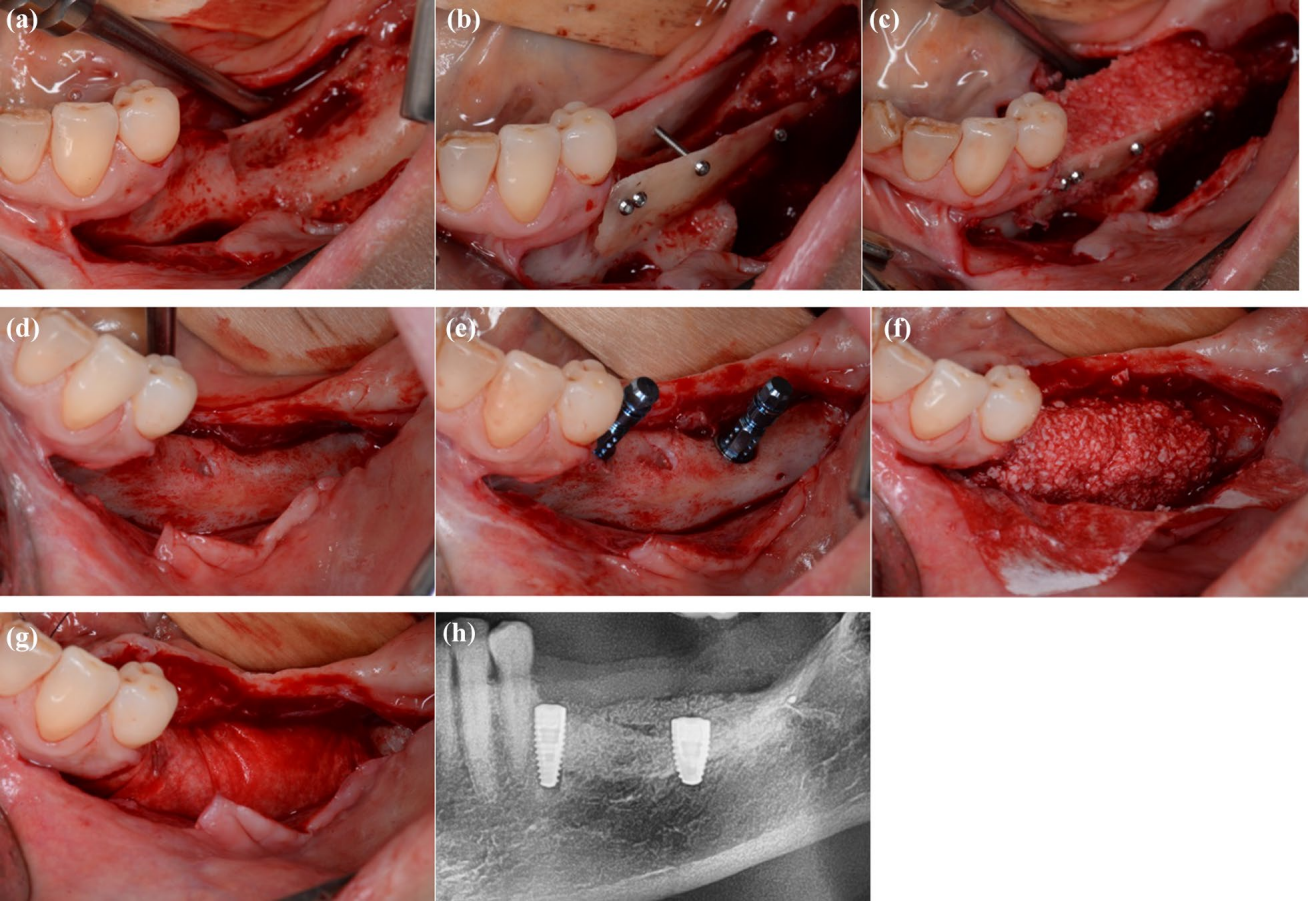
autogenous shell technique for augmentation of the posterior mandible. **a** Intraoperative site before augmentation, **b** placement of two autogenous shells, **c** filling of the gap with a mixture of allogeneic and autogenous bone, **d** site after a healing time of 4 months, **e** placement of two dental implants, **f** relining with bovine bone, **g** coverage with a collagen membrane, **h** postoperative radiograph

In brief, allogeneic and/or autogenous shells were used with allogeneic cortico-cancellous particles and/or autogenous bone chips. The autogenous bone was harvested from the same site as the recipient area or the opposite site (Table 2). There was no relationship between the number of shells used in a patient and the origin of shells. A simultaneous sinus floor elevation in the maxilla was performed in 29/117 cases (24.8%). No simultaneous dental implant placement was conducted in 115/117 patients (98.3%). In total, 229 implants were inserted 4–6 months after augmentation as planned; one patient died 2 months before implant placement. After a mean follow-up duration of 9 months, one implant was lost shortly after prosthodontic restoration (he received six implants in total after allogenic shell augmentation). This sums up to an implant survival rate of 99.6%. Figure 1a–i illustrates an allogeneic case; Fig. 2a–h shows an autogenous case.

**Table 2:** Summary of augmentation procedures

Materials		Number	Percentage
	Allogenic shell	60	51.3
	Autologous shell	52	44.4
	Allogenic and autologous shell	5	4.3
Shells per patient	1	18	15.4
	2	51	43.6
	3	29	24.8
	> 3	19	16.2
Harvesting site	Same site	37	31.6
	Opposite site	13	11.1

Total number of cases was *n* = 117

### Time of surgery

Table 3 summarizes the findings. The mean time of surgery per quadrant was 80.97 min (minimum: 24, maximum: 195; standard deviation: 34.1). Factors influencing the time of surgery were the surgeons’ experience (mean 131 (*p* < 0.001), the donor site (mean *p* < 0.001), the choice of shell material (*p* < 0.001), a simultaneous sinus floor elevation (*p* < 0.001), the occurrence of intraoperative complications (*p* = 0.004), and the number of shells (*p* < 0.001). Table 3 summarizes the respective findings. The multiple regression analyses in the reduced model showed that the factor “shell” did not influence the time of surgery (*p* = 0.128). The time required for the operation did not depend on the material used but on whether the bone shells had to be harvested. This was seen in the variable "donor site" with the values "none", "same side" and "other side" sorted in ascending order (*p* < 0.001). The new, reduced model displayed an *R*-squared of 0.727 (Table 4). In brief, surgical experience showed the most significant influence: a surgeon with more than 5 years of experience needed, on average, 51 min less for the operation than a colleague with less than 2 years of experience (*p* < 0.001). Gaining bone shells on the same side increased the operation time by 26 min. If the bone shells were harvested on the other side, the operation time increased by almost 41 min (*p* < 0.001; Table 3). The number of bone shells also increased the operation time (*p* < 0.001). Each additional shell increased the operating time by 12.5 min. If a simultaneous sinus lift was performed, this increased the active time by 19.5 min (*p* < 0.001). Intraoperative complications increased the working time by 19 min (*p* = 0.001).

**Table 3:** Results of the comparisons of the mean values of the operation time depending on various variables and their characteristic values

Variable		Number	Mean (min)	SD (min)	*p*-value
Gender	Male	34	77.26	25.1	0.454
	Female	83	82.48	37.1	
Experience	< 2 years	13	130.92	48.7	< 0.001
	> 5 years	104	74.72	26	
Donor site	None	67	68.2	24.8	< 0.001
	Same site	37	94.2	38.5	
	Opposite site	13	109	32.8	
Shell	Autologous	52	91.2	36.5	< 0.001
	Allogenic	60	69	24.7	
	Both	5	119.2	47.8	
Particles	Autologous	66	81.1	38.1	0.964
	Mixed	51	80.8	28.3	
Simultaneous implant	No	115	81.2	34.3	0.648
	Yes	2	70	14.1	
Simultaneous sinus lift	No	88	73.8	30.7	< 0.001
	Yes	29	102.7	35.2	
Intraoperative complications	No	104	77.8	32.8	0.004
	Yes	12	106.2	34.5	
Postoperative complications	No	102	79.3	33.2	0.157
	Yes	15	92.6	38.6	
Number shells	1	18	55.7	33.2	< 0.001
	2	51	76.7	24	
	3	29	85.3	36.5	
	> 3	19	109.8	33.9	

In this model (with all factors), the *R*-squared was 0.733

**Table 4:** Results of multiple regression analyses

Variable	Unstandardized coefficients	Std. Error	Standardized coefficients	*p*-value
	*B*		Beta	
Experience	− 51.3	5.5	− 0.48	< 0.001
Donor site	18.1	2.5	0.37	< 0.001
Number shells	12.5	1.9	0.35	< 0.001
Simultaneous sinus lift	19.6	4.2	0.25	< 0.001
Intraoperative complications	19	5.7	0.18	0.001

### Complications

#### Intraoperative complications

**Fig. 3:**
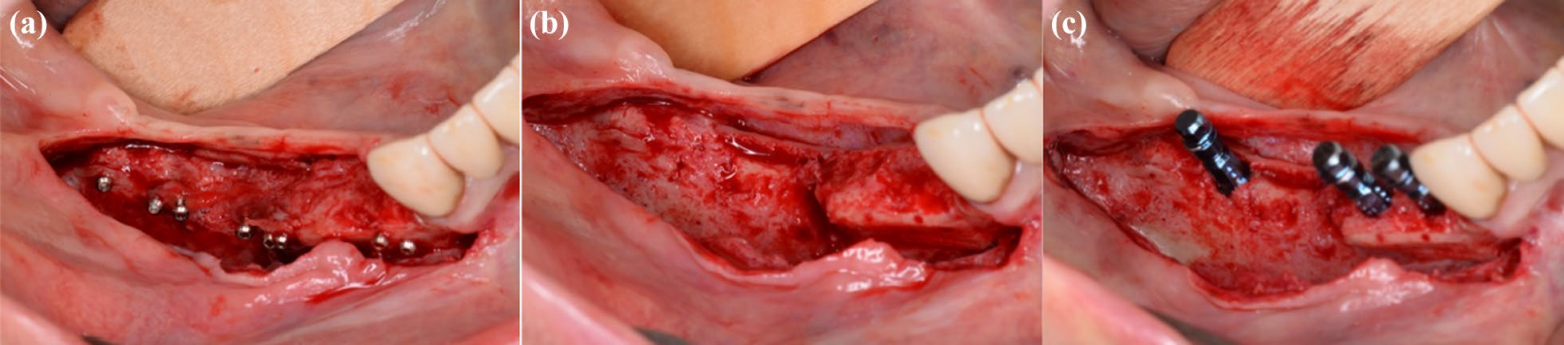
**a** Partially non-integrated bone shells during re-entry surgery after infection of the site 4 weeks after surgery, **b** same site after removal of screws, **c** placement of three dental implants was possible after all

Intraoperative minor complications were seen in 12/117 cases (10.3%). They consisted of visible sinus perforations (*n* = 5/12, 41.7%), pronounced bleeding episodes (*n* = 4/12, 33.3%), a fracture of an autogenous bone block during harvesting (*n* = 1/12, 8.3%), a fracture of an allogeneic shell (*n* = 1/12, 8.3%; Fig. 3 a-c) and severe pain after wearing of the anesthesia (*n* = 1/12, 8.3%).

#### Postoperative complications

In 3 cases each (3/117; 2.6%), a dehiscence or screw exposure occurred. A temporary sensitivity disorder was seen in 2/117 patients (1.7%). Also, a (minor) abscess had to be incised in 2/117 cases (1.7%). This summed up a postoperative complication rate of 10/117 patients (8.5%).

### Statistical analysis

There was no association between intra- and postoperative complications (*p* = 0.272). Intraoperative complications were seen significantly more often in the maxilla (*p* < 0.004) when using combinations of allogeneic and autogenous shells (*p* < 0.001), in cases of mixed particles (*p* < 0.001), and in patients of simultaneous sinus floor elevation (*p* = 0.001). In the multiple regression analyses, the combination of allogeneic and autogenous shells (95% confidence interval: 1.59–227.79; *p* = 0.02) and a simultaneous sinus floor elevation (95% confidence interval: 1.05–21.21; *p* = 0.043) showed to have a significant impact on the occurrence of intraoperative complications. For postoperative complications, no associations between the included variable were detected (all *p* > 0.05).

## Discussion

This is the first clinical study comparing allogeneic and autogenous shell augmentation regarding the time of surgery and intra- and postoperative complications. The main findings were that the time of surgery was significantly extended if the surgeon had to harvest autogenous bone either from the same or—even more—from the contralateral side. With a rate of 10.3% and 8.5%, the intra- and postoperative complications rate was low overall and is by the literature on similar procedures using allogeneic and/or autogenous bone transplants [9, 13, 30], though significantly more intraoperative complications were seen when using both allogeneic and autogenous shells and when performing a simultaneous sinus floor elevation. Besides, a less pronounced surgeons’ experience, an increase in the number of shells used, a simultaneous sinus floor elevation, and the occurrence of intraoperative complications significantly increased the time of surgery as well. The increased effort and complexity of the procedures might explain this. Also, a prolonged operative time has been reported to increase surgical site infections [27].

The efficacy of processed allogeneic bone is comparable to autogenous bone transplants [8, 13, 31–33]. An advantage of the shell technique is the combination of cortical and cancellous bone, as cancellous bone alone seems more vulnerable to bone resorption [34]. The cortical shell might be a barrier resisting external pressure [8]. Together with the sufficient bone gain and restricted implant failures reported before [12], this further proves the benefit of allogeneic shells over autogenous bone. Especially the waiving of a needed donor site—including maintaining the mandibular integrity—has to be considered. Wang et al. compared the surgical time of customized allogeneic bone blocks with autogenous bone blocks for ridge augmentation. They found a significantly shorter operation time when using allogeneic materials (mean 8.75 vs. 78.5 min) [8]. Even so, this difference might also result from the customized blocks manufactured based on the patient’s DICOM data via computer numerical control milling processes. In addition, the time needed for digital planning processes was not considered. On the contrary, in the present study, all augmentation materials had to be individually shaped and contoured before usage. Our data might more precisely reflect the time difference obtained by materials’ choice.

Though, the donor site morbidity of autogenous bone has been the focus of criticism [5]. For the cases of autogenous bone, next to an increased time of surgery, we could not give evidence for a relevant higher donor site morbidity such as nerve irritations and injuries, wound healing disturbances, bleeding, and pain. One reason might be that small-diameter shells were taken from the mandibular ramus instead of bulky blocks. In addition, the ramus of the mandible was already associated with a comparable low donor site morbidity [5]. This is to the results of others in which no donor site complications besides local swelling episodes were detected [8, 35]. For postoperative complications using allogeneic bone block grafts, Chaushu et al. reported 12.5% of soft tissue perforations, 80% of incision line opening, and 25% of block exposure. Even so, high block and implant survival rates of 92 and 100%, respectively, were seen [36]. Draenert et al. stopped their clinical study on vertical ridge augmentation with allogeneic bone blocks due to their high complication rates (implant failure 83%). Nevertheless, in this study, combined vertical and horizontal augmentations of up to 5 mm were carried out in which the implant was inserted simultaneously with the bone block in all cases. Accordingly, the high failure rate might have resulted from the technique, not the material [37]. Most other studies report meager complication rates using allogeneic materials [8, 12, 13, 35, 38].

One bias of our study might be that we do not report on long-term clinical data or implants’ survival and success rates. Even so, this was not the focus of the present investigation. Besides, next to the studies already named, the literature reports a high success rate of dental implants placed in sited augmented with allogeneic shells [12], allogeneic blocks [13, 38–41], and allogeneic granules [30, 42, 43] that are similar to those achieved when using autogenous bone only.

Even so, the effect of surgical experience—including sufficient soft tissue management—remains significant [12, 44, 45]. In our recent multicenter study on more than 300 individual cases, we describe further relevant factors and solutions for clinical success [12]. Using autogenous and/or allogeneic bone to fill the gap is also responsible for the high success rate. Khojasteh et al. reported (non-significantly) higher failure rates when using allogeneic block tenting techniques filled with bovine bone substitutes than autogenous bone [46]. We could also show that filling with bovine bone substitutes potentially leads to less bone remodeling when compared to autogenous and allogeneic particles [12].

## Conclusion

Allogeneic shell augmentation has shown the advantage of a shorter surgical time and a rate of intra- and postoperative complications similar to autogenous bone. Together with its promising clinical results, this technique can be recommended.

## Data Availability

Data are available from Jochen Tunkel upon reasonable request.

## Abbreviations

ANOVA: Analysis of variance
DBM: Mineralized processed bone allografts
DICOM: Digital imaging and communications in medicine
